# Temporomandibular Joint Prostheses: Optimal Materials for the Optimal Stomatognathic System Performance—Preliminary Study

**DOI:** 10.3390/jfb12010007

**Published:** 2021-01-26

**Authors:** Iwona Niedzielska, Michał Bąk, Damian Niedzielski, Hubert Okła, Jadwiga Gabor, Arkadiusz Stanula, Jarosław Paluch, Andrzej Szymon Swinarew

**Affiliations:** 1 Department of Cranio-Maxillo-Facial Surgery, Medical University of Silesia in Katowice, ul. Francuska 20-24, 40-027 Katowice, Poland; niedzielska.konsultant@wp.pl (I.N.); mbak@sum.edu.pl (M.B.); niedzielskidamian@gmail.com (D.N.); 2 Faculty of Science and Technology, University of Silesia in Katowice, 75 Pułku Piechoty 1A, 41-500 Chorzów, Poland; hubert.okla@us.edu.pl (H.O.); jadwiga.gabor@us.edu.pl (J.G.); andrzej.swinarew@us.edu.pl (A.S.S.); 3 Department of Individual Sports, Institute of Sport Science, The Jerzy Kukuczka Academy of Physical Education, Mikołowska 72A, 40-065 Katowice, Poland; a.stanula@awf.katowice.pl; 4 Department of Laryngology, School of Medicine in Katowice, Medical University of Silesia in Katowice, ul. Francuska 20-24, 40-027 Katowice, Poland

**Keywords:** biomechanics, prosthesis, mandibular motion, temporomandibular joint, replacement

## Abstract

The aim of this study was to quantitatively evaluate alloplastic Temporomandibular Joint (TMJ) Prostheses against other treatment modalities regarding the jaw kinematics. Six patients with Temporomandibular Joint Prostheses, four with mandibular ramus Patient-Specific Implant (PSI) with condylar head preservation, and four after mandibular condylectomy were evaluated by the means of axiography (Cadiax Compact 2), which is the noninvasive three-dimensional study of condylar movements. The patients were also evaluated clinically for the mandibular movements. The study revealed that the significant movement limitations occurred bilaterally in patients fitted with TMJ prosthesis. For the protrusion movement, the vector length of the movement (L) for the TMJ prosthesis was 0.31 vs. 3.01 mm for the PSI (Kruskal–Wallis chi-squared = 9.1667, df = 2, *p*-value = 0.01022, post hoc Dunn *p*-value = 0.015) and for the laterotrusion to the operated side, the length of the vector (L) was 0.66 vs. 3.35 mm, respectively. Statistically significant differences between groups were most frequent for the laterotrusion to the unoperated side. The study shows that a further development on TMJ Prostheses geometry and materials is needed.

## 1. Introduction

In the United States, 572 Temporomandibular Joint (TMJ) Prostheses were fitted in 2014, and one of the leading manufacturers (TMJ Concepts) produced 1004 devices. It is estimated that in 2030 those numbers will reach 902 and 1658, respectively [1]. At present, there are two FDA-approved alloplastic TMJ Total Joint Replacement (TJR) systems—these are TMJ Concepts and Zimmer Biomet. However, 27 emerging systems were identified [2].

In the systematical review, Woo-Young et al. compared the complication rate and patients Quality of Life (QoL) of costochondral rib graft versus alloplastic TMJ TJR. Seven papers were qualified, comprising 180 patients with costochondral grafts and 6 papers comprising 275 patients with alloplastic TMJ TJR. The success rate was 61% and 95%, respectively [3]. According to that, the development of TMJ TJR systems seems legit. 

The modern layout of TMJ TJR was proposed by Christensen in 1965; it consists of 2 parts, i.e., reconstructing the articular fossa (fossa component) and mandibular ramus, condyle, and head (condylar component) [4]. This started the evolution of alloplastic TMJ TJR systems. Depending on the materials used for fossa and condylar components, the devices are divided into 2 main types: Metal on Metal (MoM) and Metal on Polymer (MoP). Both were clinically tested.

In the report on 203 alloplastic TJR failures, the retrieval of the TJR due to metal hypersensitivity or device failure occurred in 33% of Christensen (MoM) devices and only in 3% of TMJ Concepts (MoP) devices [5]. Finally, the FDA approval for the MoM type Nexus/Christensen system was withdrawn and this type of prostheses is no longer in use [4]. Nowadays, the ultra-high molecular weight polyethylene (UHMWPE) is the material of choice for the fossa component manufacturing—most frequently, it is reinforced by a metal framework. The condylar components are predominantly manufactured with titanium [2]. However, even this pair of materials is not flawless. As long as the friction coefficients between titanium alloy (Ti6Al4V) and UHMWPE and between CoCr alloy (Co-Cr 28 Mo) and UHMWPE are similar, the titanium wear is significantly higher [6]. Therefore, complicated condylar titanium implants equipped with Co-Cr alloy head are used, which resolves the problem of wear. However, still the friction coefficient between Co-Cr alloy and UHMWPE is almost 50 times higher than for the cartilage-cartilage bearing couple [7].

The reconstruction with the aid of alloplastic TMJ TJR systems does not allow for the achievement of full mandible kinematics, thus is not providing the proper stomatognathic system performance. On the cadaveric simulation model, Celebi et al. showed that TMJ TJR lacks the movements of contralateral (to the TMJ TJR side) laterotrusion, protrusion, and translation during opening even when the Lateral Pterygoid Muscle (LPM) was reinserted. Furthermore, when the LPM was not reinserted, the artificial condylar head was pushed dorsal and caudal during protrusion [8]. Wojczynska et al. showed significant limitation of artificial condylar head movements during mandible opening, laterotrusion, and protrusion movements [9].

The fractures of mandibular condyle—even in the case of severe displacement—seldom cause its necrosis. This induced the researchers to scrutinize the condylar head blood supply. In the human cadaveric studies, the mixed endosteal and periosteal blood supply was reported. Moreover, it was proved that the main condylar blood supply is derived from the maxillary artery via temporal superficial and posterior deep temporal arteries and—mainly—pterygoid branches. Additionally, the blood supply comes from the transverse facial artery and masseteric artery [10,11]. Such a vascular scheme allows for the preservation of the condylar head during mandibular ramus resection. At the same time, it shows how important it is to preserve LPM attachments intact in such cases. 

The abovementioned limitations of the TMJ TJR devices inspired us to design the patient-specific implants (PSI), that allow for the preservation of the condylar head in every case it is possible. The aim of the paper is to assess the mandible kinematics depending on the reconstruction strategy. It is—how the preservation of the condylar head enhances the stomatognathic system performance.

## 2. Materials and Methods 

The study enrolled the Patients of Medical University of Silesia Maxillofacial Surgery Department who were subjected to mandibular ramus resection. The patients were divided into 3 groups: 

Group A: in which no reconstruction was performed—these patients were referred to our department after treatment in other facilities.

**Figure 1:**
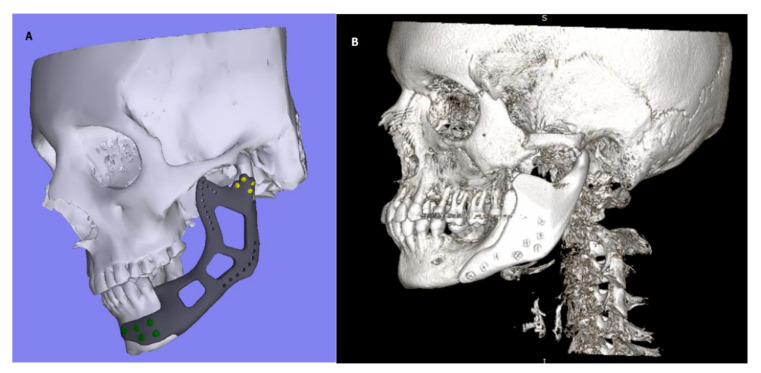
(**A**) Patient-Specific Implant (PSI) with preserved condylar head; (**B**) Temporomandibular Joint (TMJ) Total Joint Replacement (TJR) type PSI.

Group B: in which the resection was performed with the preservation of the condylar head and the reconstruction was made with titanium (Ti6Al4V) PSI. An example of such PSI is showed in Figure 1A.

Group C: in which the resection was performed without the condylar head preservation and the reconstruction was made with TMJ TJR device—that is—full TMJ prosthesis. This device consisted of titanium (Ti6Al4V) condylar component and UHMWPE fossa component. The device was individually designed and manufactured (PSI), and the design allowed for free translational movements of the artificial condyle head. An example of such PSI is showed in Figure 1B. In order to avoid bias, every patient was subjected to the same 3 phase physical therapy regime proposed by De Meurechy et al. The details on this therapy are provided in Table 1 [12]. 

In all of the groups, the axiographic evaluation with the aid of Cadiax Compact II^®^ (Gamma Medizinisch, Vienna, Austria) Mandibular Recording device was performed in order to assess the mandibular dynamic movement pattern. The device was used following the producers operating instructions. This system consists of the upper and lower face bow, registration electronic plates (flags) and markers (styli), and the central device. The upper face bow was secured by the porous knobs in the ear canals bilaterally and by the glabella support against the bridge of the nose and retained by the straps fastened behind the head. The lower face bow was securely mounted to the lower teeth with the use of an occlusal clutch and the bite registration addition silicone Occlufast Rock (Zhermack, Badia Polesine, Italy). This enabled the registration of the range of movement in protrusion, laterotrusion, and opening from the so-called reference position to the maximum range. The axiographic evaluation allows for the registration of the condylar head position in real-time with the accuracy of 0.1 mm. Moreover, the clinical evaluation with the aid of ruler and calipers was made with accuracy ranging to 1 mm. Both axiographic and clinical examinations were performed at least 1 year after surgical treatment.

The statistical analysis was performed with the aid of R 4.0.2 software (The R Foundation for Statistical Computing). In order to evaluate the differences between the groups, the Kruskal–Wallis and post hoc Dunn test were used. The significance level was set at *p* = 0.05.

The approval of the local ethic board was obtained for the study (number of the approval: KNW/0022/KB1/87/I/18).

## 3. Results

Fourteen patients were qualified for the study. Four in group A, 4 in group B, and 6 in group C. Demographics of the patients are presented in Table 2. The predominant indication for the treatment was the ameloblastoma (*n* = 5) followed by fibrous dysplasia causing dysfunction (*n* = 4) and trauma (*n* = 4). In one case, the postoperative examination revealed fibromatosis. The median age was 32.5 (range: 28–37) in group A, 47.5 (range: 33–67) in group B, and 31.5 (range: 29–42) in group C. The study enclosed 12 females and 2 males. In group B and C, the treatment was completed with the use of titanium alloy (Ti6Al4V) PSIs. The examples of such PSIs are presented in Figure 1A,B.

The results of clinical examination performed with ruler and calipers are presented in Table 3.

**Figure 2:**
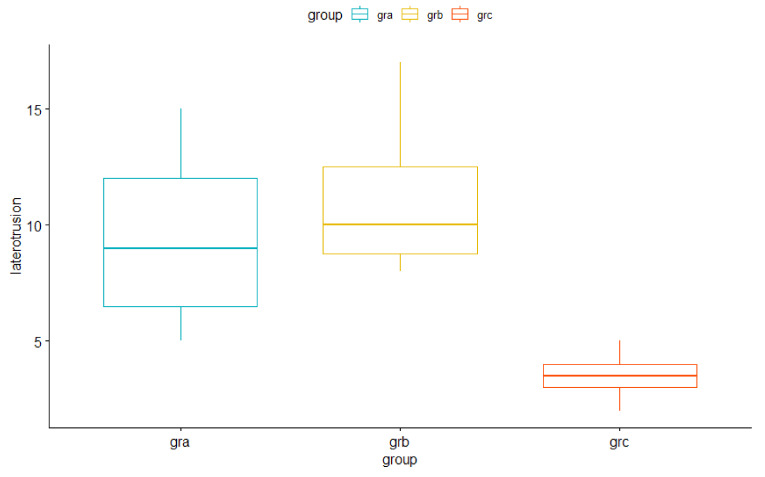
Clinical measurement of the laterotrusion to unoperated side.

Statistically significant are the differences between laterotrusion to the unoperated side (Figure 2, Kruskal–Wallis chi-squared = 9.5038, df = 2, *p* = 0.008635). The differences between group A and C and B and C are significant, and there is no significant difference between group A and B (Dunn post hoc—Table 4). It means that the clinical application of TMJ TJR was associated with significant limitation of laterotrusion to unoperated side.

**Figure 3:**
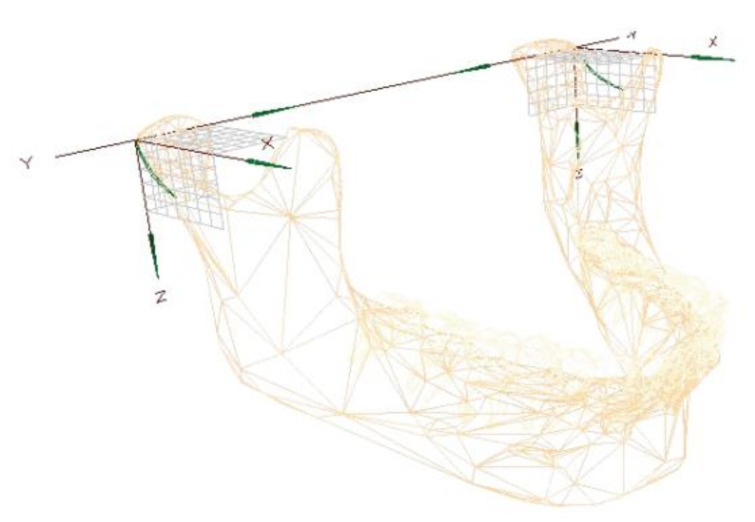
Cartesian vector components (axes X, Y, and Z) of Cadiax Compact 2.

The translation vectors of the condylar head movement were obtained from the Cadiax Compact II^®^ in a form of a Cartesian vector components in X, Y and Z axes as depicted in Figure 3. The results for the protrusion, mandible opening, laterotrusion to the operated and laterotrusion to the unoperated side are provided in the Table 5, Table 6, Table 7 and Table 8 respectively. Statistically significant differences based on the Kruskal–Wallis and post hoc Dunn tests (*p* < 0.05) were indicated with colors and—if needed—superscripted letters. For the protrusion movement, TMJ TJR were associated with lesser movement degree compared to PSIs with preserved condylar head in all 3 dimensions, which is clearly demonstrated by significantly higher vector length (L) of 3.01 s. 0.31 mm (Kruskal–Wallis chi-squared = 9.1667, df = 2, *p*-value = 0.01022, post hoc Dunn *p*-value = 0.015). For the opening movement, again group C is associated with the lesser movement degree not only for the artificial head but also for the contralateral side. For the laterotrusion to the operated side, the length of the vector (L) is shortest in group C (0.66 vs. 3.35 mm and 3.33 mm in groups B and A, respectively), which exemplifies the least movement degree in this group. Statistically significant differences between groups were most frequent for the laterotrusion to the unoperated side both for the unoperated and artificial condyle. The length of the vector (L) on the operated side was significantly longer in group B than in the other groups. Worth noticing is that in group C, the significant limitation of the condylar head movement applies also to the unoperated joint.

## 4. Discussion

When Celebi et al. applied TMJ TJR device to the cadaveric mandibular motion simulator only, the rotational movement was achieved regardless of the LPM reattachment. Moreover, the laterotrusion to the unoperated side was unobtainable and the condylar head was moving dorsally on the operated side during the protrusion when the LPM was not reattached [8]. Wojczynska et al. reported that the movement of the condylar head after TMJ TJR is significantly decreased [9]. On the other hand, the same author describes 4 cases, in which after implantation of the alloplastic TMJ Concepts devices, the extended range of movement was obtained on the operated side [13]. Moreover, Mommaerts reported on establishing up to 62.5% of preoperative laterotrusion and 40% of normal laterotrusion after TMJ TJR with LPM reinsertion [14]. Those results do not go in line with the abovementioned results on the cadaveric motion simulator [8]. Nevertheless, in the latter, the stock TMJ Concepts device was used, while in the Mommaerts et al. study, the PSIs were used. These results show that to obtain the best possible range of motion, which provides the proper stomatognathic system performance, it is important not only to preserve the attachment and function of LPM but also to obtain the proper position and geometry of the TMJ TJR device. The translation movement in healthy joint ranges up to 16 mm. Despite alloplastic TMJ TJR devices allow for this type of movement, it is in vivo significantly limited. Up to date, many TMJ TJR devices were presented, which are known for the limited range of the translational movement [15]. Van Loon et al. proposed the theoretical foundations for the TMJ prosthesis with a lowered center of rotation (CR), which imitates the translational movement [16]. The further works allowed for the creation of the endoprosthesis with lowered CR—the Groningen prosthesis. Initially, it functioned as the stock prosthesis but, nowadays, was adopted as the PSI [15]. The limited translational movement of the TMJ TJR devices is attributed to tissue stiffness, scar formation, and a relatively high friction coefficient in artificial joints [16]. The friction coefficient for Co-Cr and UHMWPE bearing couple is almost 50 times higher than for the cartilage-cartilage couple (Table 9) [7].

There are three possible strategies to face this problem. One is the modification of the polymer material, and second is the modification of the metallic surface. In addition, the attempts to develop the lubricants for the artificial joints were made [6].

In the presented research, it was unequivocally exhibited that when the natural articular TMJ surfaces were preserved, the range of motion was higher both on the operated and unoperated side, even when the LPM attachment was partially lost. It may be attributed—among others—to the relatively low friction coefficient in the natural joint. The abovementioned literature data show that obtaining the ideal condylar head movement after TMJ TJR is most probably the matter of achieving the optimal endoprosthesis geometry and material properties, preserving as many bony structures as possible and preserving or re-establishing LPM attachment. In the presented research it was unequivocally exhibited that when the natural articular TMJ surfaces were preserved, the range of motion was higher both on the operated and unoperated side, even when the LPM attachment was partially lost. It may be attributed—among others—to the relatively low friction coefficient in the natural joint. The abovementioned literature data show that obtaining the ideal condylar head movement after TMJ TJR is most probably the matter of achieving the optimal endoprosthesis geometry and material properties, preserving as many bony structures as possible and preserving or re-establishing LPM attachment.

## 5. Conclusions

The acquired results unequivocally show that even the partial restoration of LPM function and preservation of condylar head significantly enhance the stomatognathic system performance. Therefore, whenever the condylar head preservation is possible, it should be performed, because the proposed approach allows for the better and more natural freedom of movement of bilateral TMJs. Moreover, worth noting is that alloplastic TMJ TJR decreases the range of motion of both condylar heads including the natural contralateral condylar head, thus affecting the function of both TMJs. Additionally, the discovery that the preservation of the natural articular TMJ surfaces regardless of the preservation of the LPM attachment allows for the best freedom of movement has led to the conclusion that the endoprosthesis geometry and material properties may play more important role in the functional outcomes than preserving the LPM attachments. This finally, shows where the focus should be put on in the TMJ TJR devices development. 

## Figures and Tables

**Table 1:** Rehabilitation schedule proposed by De Meurechy et al. [12]**.**

Phase	Timing	Therapy
Phase 1	Within 24 hr after surgery to 7 days after surgery	Non-chewing diet
		Cold therapy over joint 1 × 20 minutes (minimally), 5 times per day
		Condylar rotational exercises (passive and active opening and closing, 20 repetitions, 3 times/day; active opening and closing, 20 repetitions, 3 times/day)
		Grade I joint distraction
		Grade II joint distraction toward end of phase 1
		Oral re-education with avoidance of parafunctions
Phase 2	1–3 wk after surgery	Soft diet
		Moist heat application over muscles for 20 minutes before exercises, cold application over joint after exercises
		Coordination exercise using a mirror
		1. Condylar rotational exercises as in phase 1
		2. Active mouth opening and closing
		3. ‘Mandibular snake’: protrusion, depression, retrusion, elevation, return to neutral position
		Range of motion exercises (until pain limit, not over pain limit)
		1. Insertion of tongue spatula or TheraBite system 7 × 7 seconds, 7 times/day
		2. Active assisted opening: 10 repetitions, keeping maximal mouth opening for 30 seconds, 3 times/day
		3. Active lateral movement: 10 repetitions, keeping maximum lateral deviation for 30 seconds, 3 times/day
		4. Active protrusive and retrusive movement: 10 repetitions, keeping pro- and retrusive deviation for 30 seconds, 3 times/day
		Grade II joint distraction
		Use of chewing gum
Phase 3	From 4 wk onafter surgery	Transition to solid diet
		Stabilization exercises
		1. Lower jaw maintained in neutral, slightly open position (lateral manual pressure: 1 × 6 repetitions, 5 times/day; upward manual pressure: 6 repetitions, 5 times/day)
		2. Lower jaw maintained in closed position (attempting to open during upward manual pressure: 6 repetitions, 5 times/day)
		Range-of-motion exercises
		1. Maximum opening (insertion of tongue spatula or TheraBite system: 5 × 30 seconds, 5 times/day; active assisted opening: 5 repetitions, keeping maximum mouth opening for 30 seconds to 1 minute, 3 times/day; active opening: 5 repetitions, keeping maximum mouth opening for 30 seconds to 1 minute, 3 times/day)
		2. Lateral deviation: 10 repetitions, 3 times/day per side
		Grade III and grade IV joint distraction
		Massage of masticatory muscles
		Use of chewing gum

Reprinted from Journal of Oral and Maxillofacial Surgery Volume 77, Issue 5 Nikolas K.G. De Meurechy, Pieter-Jan Loos, and Maurice Y. Mommaerts “Postoperative Physiotherapy After Open Temporomandibular Joint Surgery: A 3-Step Program” pages 932-950, 1 May 2019 with permission from Elsevier.

**Table 2:** Patients demographics.

	Group A (*n* = 4)	Group B (*n* = 4)	Group C (*n* = 6)
Sex:	-	-	-
F	4 (100%)	4 (100%)	4 (66.6%)
M	0	0	2 (33.3%)
Median age (years)	32.5 (range: 28–37)	47.5 (range: 33–67)	31.5 (range: 29–42)
Indication for treatment:	-	-	-
Ameloblastoma	0	4 (100%)	1 (16%)
Fibrous dysplasia	2 (50%)	0	2 (33.3%)
Trauma	2 (50%)	0	2 (33.3%)
Fibromatosis	0	0	1 (16%)

**Table 3:** Clinical examination results.

	Group A (*n* = 4)	Group B (*n* = 4)	Group C (*n* = 6)
Opening pattern:	-	-	-
straight	-	2 (50%)	-
deviated	4 (100%)	2 (50%)	6 (100%)
Opening (mm) (median)	43.5 (range: 35–50)	37.5 (range: 30–42)	36 (range: 30–40)
Laterotrusion to operated side (mm) (median)	1.25 (range: 1–2)	4.5 (range: 2–6)	1 (range: 0–2)
Laterotrusion to unoperated side (mm) (median)	9 (range: 5–15) *	10 (range: 8–17) *	3.5 (range: 2–5) *
Protrusion (median)	4 (range: 2–6)	5.5 (range: 3–8)	2.5 (range: 1–7)

* Statistically significant differences Kruskal–Wallis chi-squared = 9.5038, df = 2, *p* = 0.008635.

**Table 4:** Post hoc Dunn test for laterotrusion to unoperated side.

	Group A	Group B
Group B	0.641	–
Group C	0.044	0.015

**Table 5:** Mean cartesian vector components and mean vector lengths for protrusion movement. Data are presented as mean ± SD, X, Y, Z—cartesian vector components, L—vector length. Statistically significant differences (Kruskal–Wallis and post hoc Dunn tests, *p* < 0.05) were indicated with colors.

Group		Group A		Group B		Group C	
Side		Operated	Unoperated	Operated	Unoperated	Operated	Unoperated
variable	X	0.42 ± 0.38	0.85 ± 0.66	−1.31 ± 1.27	1.51 ± 0.83	−0.662 ± 1.11	0.17 ± 0.25
	Y	−0.14 ± 0.07	−0.21 ± 0.1	−0.91 ± 0.45	−0.69 ± 0.3	0.12 ± 0.16	0.06 ± 0.13
	Z	−1.59 ± 0.51	1.23 ± 1.45	−1.97 ± 1.51	2.5 ± 1.23	0.03 ± 0.18	−0.13 ± 0.22
	L	1.81 ± 0.63	1.97 ± 1.08	2.82 ± 1.59	3.01 ± 1.5	0.74 ± 1.08	0.31 ± 0.27

**Table 6:** Mean cartesian vector components and mean vector lengths for mandible opening. Data are presented as mean ± SD, X, Y, Z—cartesian vector components, L—vector length. Statistically significant differences (Kruskal–Wallis and post hoc Dunn tests, *p* < 0.05) were indicated with colors and superscripted letters if needed.

Group		Group A		Group B		Group C	
Side		Operated	Unoperated	Operated	Unoperated	Operated	Unoperated
variable	X	1.61 ± 1.48	1.35 ± 1.01	−1.07 ± 1.22	2.88 ± 0.76	−0.18 ± 0.2	0.55 ± 1.25
	Y	0.08 ± 0.25	0.01 ± 0.17	−1.07 ± 0.39	−0.77 ± 0.36	−0.12 ± 0.17	−0.24 ± 0.19
	Z	−1.48 ± 0.18 ^a^	−1.05 ± 0.53	−2.14 ± 0.82 ^b^	4.35 ± 1.01	−0.55 ± 0.58 ^a,b^	1.43 ± 2.69
	L	2.56 ± 0.6 ^a^	1.88 ± 1.07	2.76 ± 1.25 ^b^	5.28 ± 1.3	0.68 ± 0.53 ^a,b^	2.55 ± 1.98

**Table 7:** Mean cartesian vector components and mean vector lengths for laterotrusion to the operated side movement. Data are presented as mean ± SD, X, Y, Z—cartesian vector components, L—vector length. Statistically significant differences (Kruskal–Wallis and post hoc Dunn tests, *p* < 0.05) were indicated with colors and superscripted letters if needed.

Group		Group A		Group B		Group C	
Side		Operated	Unoperated	Operated	Unoperated	Operated	Unoperated
variable	X	1.84 ± 1.41	0.68 ± 0.37	−1.73 ± 0.68	2.11 ± 0.91	−0.3 ± 0.33	0.58 ± 0.9
	Y	0.28 ± 0.17	0.06 ± 0.07	−1.10 ± 0.4	−0.8 ± 0.33	−0.26 ± 0.24	−0.33 ± 0.25
	Z	−2.62 ± 1.03 ^a^	1.66 ± 1.6	−2.63 ± 0.63 ^b^	3.21 ± 1.37	−0.52 ± 0.3 ^a,b^	1.39 ± 2.05
	L	3.33 ± 1.58 ^a^	2.13 ± 1.18	3.35 ± 0.95 ^b^	3.92 ± 1.68	0.66 ± 0.43 ^a,b^	2.03 ± 1.7

**Table 8:** Mean cartesian vector components and mean vector lengths for laterotrusion to the unoperated side movement. Data are presented as mean ± SD, X, Y, Z—cartesian vector components, L—vector length. Statistically significant differences (Kruskal–Wallis and post hoc Dunn tests, *p* < 0.05) were indicated with colors and superscripted letters if needed.

Group	Group A		Group B		Group C	
Side	Operated	Unoperated	Operated	Unoperated	Operated	Unoperated
X	1.76 ± 1.3	0.26 ± 0.23 ^a^	−1.51 ± 0.52	2.15 ± 0.42 ^a,b^	−0.59 ± 0.65	−0.04 ± 0.56 ^b^
Y	0.18 ± 0.33 ^a^	−0.2 ± 0.03	−1.07 ± 0.13 ^a,b^	−0.76 ± 0.1	0.1 ± 0.27 ^b^	0.08 ± 0.13
Z	−1.71 ± 0.22	0.22 ± 0.87 ^a^	−2.45 ± 0.33	3.25 ± 0.64 ^a,b^	−0.128 ± 0.51	0.24 ± 1.28 ^b^
L	2.68 ± 0.9 ^a^	1.06 ± 0.57 ^b^	3.08 ± 0.53 ^a,c^	3.97 ± 0.77 ^b,d^	0.88 ± 0.52 ^c^	1.21 ± 0.56 ^d^

**Table 9:** Friction coefficient for bearing couples used in Temporomandibular Joint (TMJ) Total Joint Replacement (TJR). Adopted from Basu B. Advanced Biomaterials: Fundamentals, Processing, and Applications. Hoboken, N.J. Westerville, Ohio: John Wiley & Sons American Ceramic Society; 2009 [7].

Bearing Couples	Coefficient of Friction
Cartilage-cartilage	0.002
CoCr-UHMWPE	0.094
Zirconia-UHMWPE	0.09–0.11
Alumina-UHMWPE	0.08–0.12
CoCr-CoCr	0.12
Alumina-alumina	0.05–0.1

## Data Availability

The raw data obtained from the Cadiax Compact II^®^ system is available at https://drive.google.com/file/d/15SlzuPzgPLF2rtT4cXy6PEeE68ff6eTM/view?usp=sharing.
